# Dopamine regulates termite soldier differentiation through trophallactic behaviours

**DOI:** 10.1098/rsos.150574

**Published:** 2016-02-10

**Authors:** Hajime Yaguchi, Takaya Inoue, Ken Sasaki, Kiyoto Maekawa

**Affiliations:** 1 Graduate School of Science and Engineering, University of Toyama, Toyama, Japan; 2 Department of Bioresource Science, Tamagawa University, Tokyo, Japan

**Keywords:** caste polyphenism, dopamine, soldier differentiation, proctodeal trophallaxis

## Abstract

Caste polyphenism in social insects is regulated by social interactions among colony members. Trophallaxis is one of the most frequently observed interactions, but no studies have been conducted identifying the intrinsic factors involved in this behaviour and caste differentiation. Dopamine (DA) has multiple roles in the modulation of behaviours and physiology, and it produces species-specific behaviours in animals. Here, to verify the role of DA in termite soldier differentiation, we focused on the first soldier in an incipient colony of *Zootermopsis nevadensis*, which always differentiates from the oldest 3rd instar (No. 1 larva) via a presoldier. First, brain DA levels of the No. 1 larva at day 3 after its appearance were significantly higher than day 0. Second, DA synthesis gene expression levels were extraordinarily high in the No. 1 larva at day 0–1 after appearance. Finally, injection of a DA receptor antagonist into the No. 1 larva resulted in the inhibition of presoldier differentiation. Behavioural observations of the antagonist or control-injected larvae suggested that brain DA and signalling activity regulate the frequencies of trophallaxis from reproductives and presoldier differentiation. Because trophallaxis is a social behaviour frequently observed in natural conditions, the role of DA should be investigated in other social insects with frequent trophallactic and allogrooming behaviour.

## 1. Background

Phenotypic plasticity, which is defined as the ability to express adaptive phenotypes in response to variable environmental conditions from a single genotype, may drive adaptive evolution [1]. Polyphenism is an extreme case of phenotypic plasticity, and alternative distinct phenotypes are produced depending on extrinsic factors. Polyphenism normally involves an alteration in behaviours related to life history as well as morphological traits, and most examples can be observed in insects, such as the phase transition in locusts, the winged and wingless forms in aphids and the caste differentiation in social insects [2]. Although there are several important studies on the neurochemical basis of behavioural changes in polyphenisms [3,4], the regulatory mechanisms of behavioural changes accompanying the morphological alterations are still unknown.

In a nest of termites, there are different types of individuals (castes) including reproductives (queen and king), workers and soldiers. Termites are one of the major groups of social insects and have well-organized colonies with cooperation among the castes. Caste differentiation in termites is essentially determined in response to extrinsic factors, including chemical substances transferred by social interactions between colony members, during the postembryonic period [5]. Termite soldiers are a unique caste among social insects in terms of their specific weapon morphology and defensive behaviours [6]. All extant species (except for some lineages with secondary loss) have a soldier caste, which strongly suggests that soldiers in termites evolved just once in the course of eusocial evolution [7,8]. Moreover, it is thought that soldiers were first acquired among sterile castes in the initial steps in social evolution, because there were increasing needs for reproductives not only to defend the nest but also to devote themselves to reproduction [9]. Consequently, revealing the mechanisms of termite soldier differentiation is one of the most important challenges in the comprehensive understandings of eusocial evolution in social insects.

Termite soldiers differentiate from workers by two moults via a presoldier stage, and this change requires high juvenile hormone (JH) titre in workers [5,10]. Previous studies reported that artificial presoldier differentiation in response to JH or JH analogue treatments applied to workers was successful in many termite species. Using these techniques, some important molecules during presoldier differentiation after an increase in JH titre in workers have been elucidated [11–17]. Generally, in termites, additional soldier differentiation is inhibited by existing soldiers in the nest, probably because of the presence of negative feedback pheromone regulation (reviewed in [5]). On the other hand, soldier differentiation is promoted by the existence of reproductives in some species (reviewed in [5]). Thus, the first soldiers apparently develop in incipient colonies in response to reproductive cues and their presence inhibits the formation of more soldiers [18]. However, the mechanisms underlying the JH increase in workers remain to be determined. In this study, we focused on presoldier differentiation in natural conditions to clarify these mechanisms. As far as we are aware, there is only one experimental system that can identify a worker destined to be a soldier, which occurs in the damp-wood termite *Zootermopsis nevadensis* Hagen (Isoptera: Archotermopsidae). In an incipient colony of this species, the oldest 3rd instar larva (first moulted 3rd instar larva; No. 1 larva) always differentiates into a presoldier within 7–8 days, and the next-oldest 3rd instar larva (second moulted 3rd instar larva; No. 2 larva) moults into a 4th instar worker within 20 days (electronic supplementary material, figure S1) [18,19]. Furthermore, behavioural differences were observed between these larvae. Namely, the No. 1 larvae frequently received proctodeal trophallaxis from the reproductives (queen and king), but the No. 2 larvae spent significantly more time allogrooming colony members. Because this is the first case to show that a particular individual differentiates into a presoldier before the moult, the regulatory mechanisms of the behavioural characteristics observed in the No. 1 larvae may be crucial for presoldier differentiation.

Biogenic amines are metabolic products from amino acids, which are conserved extensively across animal taxa, and these substances work as neurotransmitters, neuromodulators and neurohormones in the central nervous systems (CNS) [20,21]. In particular, dopamine (DA), which is synthesized from tyrosine (electronic supplementary material, figure S2), is known as an important factor for mediating several behavioural changes in insects. For example, in the American cockroach *Periplaneta americana*, the duration of grooming was decreased by the injection of a DA receptor antagonist into adult males [22]. In the migratory locust *Locusta migratoria*, transitions from gregarious to solitary behaviour were induced by the injection of a DA receptor antagonist into gregarious nymphs [23]. These findings indicated that DA in nervous systems regulates behavioural alterations in a species-specific manner. Moreover, there are some reports on the roles of DA in hymenopteran social insects. For example, in the honeybee *Apis mellifera*, tyrosine-fed or royal jelly-fed workers had higher brain DA levels than sucrose-fed individuals, resulting in ovarian development and the inhibition of foraging in the former [24]. In the ponerine ant *Diacamma* sp., DA was increased in the brain in queen-destined individuals compared with worker-destined individuals, and it could promote ovarian development during social competition [25]. In the ant *Formica japonica*, brain DA levels of starving and sucrose-fed individuals were elevated by social interactions with nest-mates via trophallaxis [26]. Thus, DA can regulate the behavioural and physiological transition between castes and may be associated with trophallaxis. Because termite soldier differentiation is mediated by trophallactic interactions [18], DA might be involved in this differentiation, via social interactions, for example, specific trophallactic behaviours observed in the No. 1 larvae of *Z. nevadensis*.

To test this hypothesis, we performed the following experiments using the No. 1 and No. 2 larvae in an incipient colony of *Z. nevadensis*. First, DA levels in the larval brains were measured. Second, the expression levels of DA biosynthetic and receptor genes in these larvae were quantified. Finally, we examined whether the injection of a DA receptor antagonist influenced the frequency of social trophallactic behaviours and presoldier differentiation rates.

## 2. Material and methods

### 2.1 Termite collection and incipient colony foundation

Mature colonies of *Z. nevadensis* were collected from Kawanishi-shi, Hyogo Prefecture, Japan, in April 2011, October 2011 and April 2013. Decayed logs containing a large number of nymphs were cut off and brought to the laboratory. They were placed in plastic boxes and maintained in constant darkness at room temperature until alates emerged from the colonies. Alates were sampled and separated by sex based on the configuration of the genital plate [27]. In accordance with the methods of previous studies [18,28], male and female alates from different colonies were paired in 60 mm plastic dishes with crushed pieces of nest wood. These dishes were kept in constant darkness at 25°C.

### 2.2 Measurements of dopamine and its metabolite

In accordance with a previous study [18], the oldest 3rd instar (No. 1 larva) and the second larva that moulted into a 3rd instar (No. 2 larva) were marked with different coloured waterproof inks. The head width of the larval instar was measured based on a previous study [29]. The No. 1 and No. 2 larvae were collected at days 0 and 3 after their appearance (*n*=12 (No. 1 larvae at day 0 and 3), 10 (No. 2 larvae at day 0) and 14 (No. 2 larvae at day 3)). Collected individuals were immersed immediately in liquid nitrogen and stored therein using a storage container DR-3 (Cryo one, Osaka, Japan) until use. Within one month, measurements of DA and *N*-acetyldopamine (NADA, a metabolite of DA; electronic supplementary material, figure S2) levels in the brains were performed based on a previous study [30]. Frozen individuals were separated into heads and other body parts on ice, and brains were isolated from the heads in ice-cold saline (128.33 mM NaCl, 2.68 mM KCl, 1.80 mM CaCl_2_, pH 6.7) within 3 min. Each sample was pooled from two individuals because of the small size of larval brain. Brains were added into 50 μl of 0.1 M perchloric acid solution containing 12.5 ng ml^−1^ 3,4-dihydroxybenzylamine (DHBA) (Aldrich, Milwaukee, WI, USA) and immediately homogenized using a microglass homogenizer (Wheaton, Millville, NJ, USA). These homogenized solutions were centrifuged at 20 000*g* for 30 min at 4°C and supernatants were collected for high-performance liquid chromatography with electrochemical detection (HPLC-ECD) analysis. The system consisted of a solvent delivery pump, a refrigerated automatic injector and a C18 reverse-phase column UG 120 (Shiseido, Tokyo, Japan) maintained at 35°C in a column oven. An electrochemical detector ECD-300 (Eicom, Kyoto, Japan) with a glassy carbon electrode was set at 0.8 V. The mobile phase contained 0.18 M monochloroacetic acid, 40 μM Na_2_EDTA (Wako, Osaka, Japan) adjusted to pH 3.6 using NaOH (Wako). Sodium 1-octanesulfonate (1.62 mM) (Nacalai Tesque, Kyoto, Japan) and CH_3_CN (Nacalai) (final concentration 7.4%, v/v) were added to this solution. After preparing the solution, the mobile phase was degassed for 15 min. External standards (DA, NADA (Sigma, St Louis, MO, USA)) and DHBA (Aldrich) were used in this analysis for the identification and quantification of DA and NADA. The levels of these amines were quantified from the areas of peaks in the chromatogram obtained by HPLC-ECD using data analysis software PowerChrom (ADInstrument, NSW, Australia).

### 2.3 Gene expression analysis

#### 2.3.1 Identification of dopamine-related genes

Terrapon *et al.* [31] reported genes involved in DA biosynthesis and receptors, and the following six genes were obtained from the database [31] (http://termitegenome.org/): tyrosine hydroxylase (TH), dopa decarboxylase (DDC), *N*-acetyltransferase (NAT), DA receptor 1 (DopR1), DA receptor 2 (DopR2) and DA receptor 3 (DopR3). TH converts tyrosine into L-DOPA, and DDC catalyses DA synthesis from L-DOPA in the next step (electronic supplementary material, figure S2). DA receptor genes belonging to the superfamily of G-protein coupled receptors are broadly conserved among insects [21,32]. For gene expression analysis, specific primers were newly designed using Primer 3 plus [33] (electronic supplementary material, table S1).

#### 2.3.2 Total RNA extraction and cDNA synthesis

The No. 1 and No. 2 larvae were collected from each incipient colony at three time points (0–1, 2–3 and 4–5 days) after their appearance. Eighteen different individuals were collected at each time point, and a total 54 individuals were used for the analysis. Collected individuals were immersed immediately in liquid nitrogen and stored at −80°C until use. Three different individuals per sample were pooled, and each sample was prepared in biological sextuplicates. Total RNA was extracted from whole bodies using ISOGEN (NipponGene, Tokyo, Japan). The extracted total RNA was purified using recombinant DNase I (Takara, Shiga, Japan) to remove genomic DNA. RNA purity and quantity were measured using a NanoVue spectrophotometer (GE Healthcare BioSciences, Tokyo, Japan). Equal concentrations of total RNA (215 ng) were reverse transcribed for cDNA synthesis using a high-capacity cDNA Reverse Transcription Kit (Applied Biosystems, Foster City, CA, USA). Real-time quantitative PCR was performed using a Thunderbird qPCR mix (Toyobo, Osaka, Japan) with a MiniOpticon real-time PCR detection system (Bio-Rad, Hercules, CA, USA). The suitability of six reference genes as internal control genes (*EF*-*1*α**, *β*-*actin*, *NADH*-*dh*, *RS49*, *RPS18* and *RPL13*α**) of *Z. nevadensis* (GenBank accession nos. AB915828, AB915826, AB936819, KDR21989, KDR22651 and KDR22610, respectively) was evaluated using software GeNorm [34] and NormFinder [35]. The qPCR analysis was performed in biological sextuplicates.

### 2.4 Pharmacological analysis

#### 2.4.1 Injection of dopamine receptor antagonist

Just after the No. 1 larva emerged, reproductives (queen and king) and the No. 1 larva were transferred to a 46 mm Petri dish containing a moistened filter paper (Advantec, Tokyo, Japan). After 24 h (day 1 after the appearance of the No. 1 larva), the No. 1 larvae were placed on ice for 90 s to reduce movement, and an equal volume (32.2 nl) of double-distilled water (DDW) as a control or DA receptor antagonist, *cis*-(Z)-flupenthixol dihydrochloride (MP Biomedical, Santa Ana, CA, USA), diluted with DDW (100 pM and 1 nM) was injected using Nanoliter 2000 (World Precision Instruments, Sarasota, FL, USA) using a glass capillary (10–30 μm diameter) (DDW (*n*=9), DA antagonist 100 pM (*n*=10) and 1 nM (*n*=10)). Each solution was coloured with Fast Green FCF (Tokyo Chemical Industry, Tokyo, Japan), and injection within the body was confirmed. Each dish with the solution-injected No. 1 larva and reproductives was maintained in constant darkness at 25°C. On the lid of each dish, a cross was drawn for the observation of locomotor activity (see below). The female reproductive (queen) was marked on her abdomen with waterproof ink.

#### 2.4.2 Observations of behaviours and presoldier differentiation

Behavioural observation was performed in accordance with a previous study [18]. Each dish was placed in constant darkness for 30 min at room temperature before video recording. Social behaviours (proctodeal trophallaxis and allogrooming) and locomotor activity of the No. 1 larvae were recorded for 5 days (40 min per day) using a CX1 (Ricoh, Tokyo, Japan) digital camera. The frequencies of proctodeal trophallactic behaviour from reproductives to the No. 1 larva, and those of allogrooming behaviour from the No. 1 larva to reproductives were counted. We confirmed that both behaviours continued for more than 3 s. When proctodeal trophallactic behaviour was observed within 1 min after allogrooming behaviour, we omitted the count of allogrooming behaviour (namely, 1 proctodeal trophallaxis and 0 allogrooming), because proctodeal trophallaxis was normally started by allogrooming-like stimulation of the donor's perianal region by the recipient [36]. Locomotor activity was measured as the frequencies of intersection of the cross drawn on the lid of each dish. After video recording, all dishes were kept in constant darkness at 25°C, and checked every 24 h to determine whether the No. 1 larva had moulted into a presoldier or a 4th instar worker.

### 2.5 Statistical analysis

Brain levels of DA and NADA in each larva were compared between day 0 and 3 after appearance using Welch's *t*-test (*p*<0.05). Expression levels of DA-related genes were compared among three time points (0–1, 2–3 and 4–5 days after appearance) using one-way ANOVA followed by *post hoc* multiple comparison test (Tukey's test, *p*<0.05). Behavioural frequencies were compared for each individual using one-way ANOVA followed by Tukey's test (*p*<0.05). All of these tests were performed using Mac Statistical Analysis v. 2.0 (Esumi, Tokyo, Japan). Prior to the use of one-way ANOVA, we performed Levene's test on variance equality using R v. 2.15.2 [37]. Presoldier differentiation rates were compared between the DDW- and antagonist-injected individuals by Ryan's test using R v. 2.15.2.

## 3. Results

### 3.1 Brain dopamine and *N*-acetyldopamine levels

**Figure 1.**
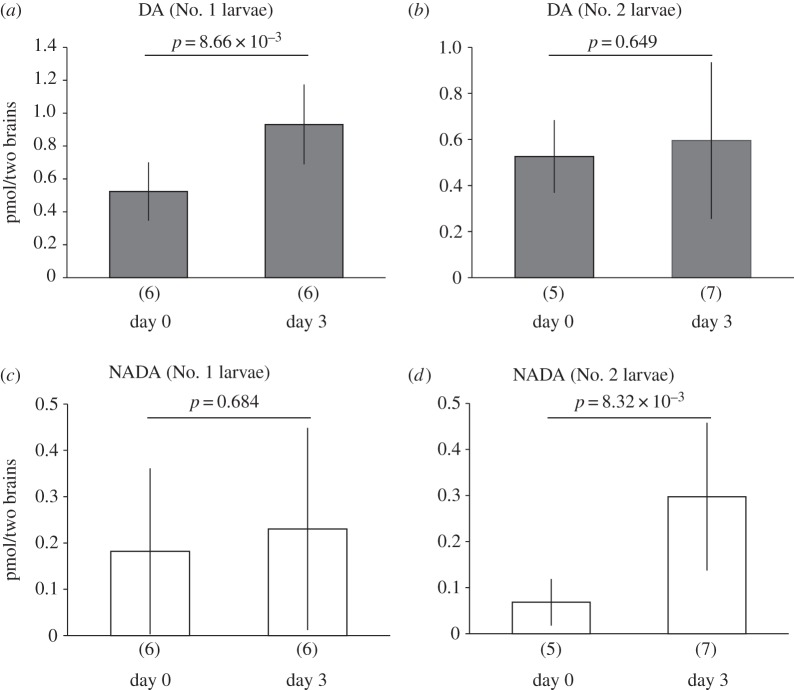
Brain dopamine (DA) and *N*-acetyldopamine (NADA) levels (mean ± s.d.) in the No. 1 (*a*,*c*) and No. 2 larvae (*b*,*d*) between day 0 and 3 after appearance. Sample numbers examined are shown in parentheses. Each sample was pooled from two individuals. *p*-values (Welch's *t*-test) are shown above the bars.

Brain DA levels in the No. 1 larvae at day 3 after appearance were significantly higher than those of day 0 (figure 1*a*; Welch's *t*-test, *p*<0.01). On the other hand, those in the No. 2 larvae were not significantly different between day 0 and 3 after the appearance (figure 1*b*; *p*=0.649). Brain levels of NADA, a DA metabolite, in the No. 1 larvae were not significantly different between days 0 and 3 (figure 1*c*; *p*=0.684). However, those in the No. 2 larvae at day 3 were significantly higher than those at day 0 (figure 1*d*; *p*<0.01).

### 3.2 Expression levels of dopamine-related genes

Expression analyses were performed by real-time quantitative PCR using the No. 1 and No. 2 larvae collected at three time points (0–1, 2–3 and 4–5 days after appearance). Because both GeNorm and NormFinder software confirmed that the expression levels of *RPL13*α**were the most stable, we used it as the reference gene. Expression levels of *TH*, *DDC* and *NAT* at day 0–1 were significantly higher in the No. 1 larvae, but significant differences were not observed at days 2–3 and 4–5 (electronic supplementary material, figure S3*a*–*c*; one-way ANOVA followed by Tukey's test, *p*<0.05). In the No. 2 larvae, the expression patterns of these genes were different from those observed in the No. 1 larvae. Expression levels of *TH* at day 0–1 were significantly higher than those at day 4–5 (electronic supplementary material, figure S3*d*). However, *DDC* expression levels at day 4–5 were significantly higher than those at day 0–1 (electronic supplementary material, figure S3*e*). Expression levels of *NAT* were not significantly different among the three time points (electronic supplementary material, figure S3*f*). Expression levels of three DA receptor genes were not significantly different among three time points both in the No. 1 and No. 2 larvae (electronic supplementary material, figure S4).

### 3.3 Effects of injection of dopamine receptor antagonist

#### 3.3.1 Presoldier differentiation rate

**Figure 2.**
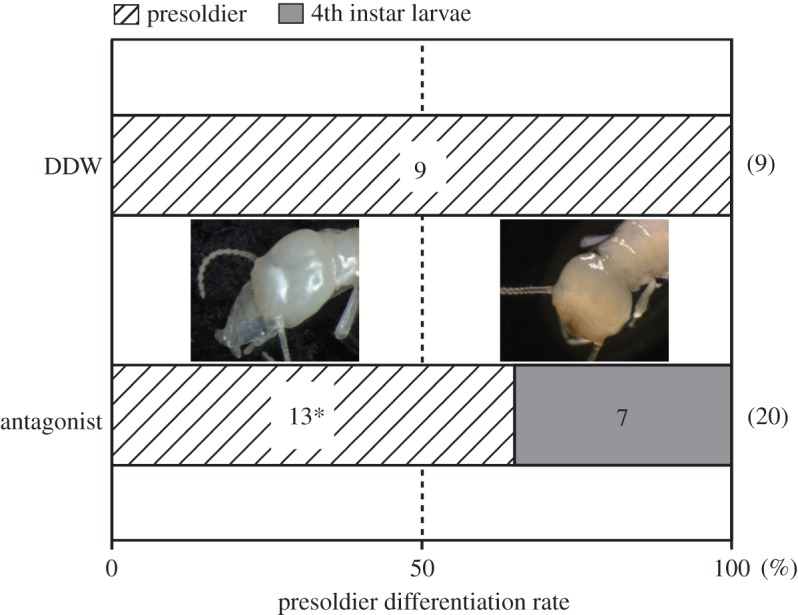
Presoldier differentiation rates from the solution-injected No. 1 larvae. Numerical values in the columns represent the numbers of individuals that moulted into presoldiers or 4th instar larvae. Numbers examined are shown in parentheses. Asterisk indicates a significant difference (Ryan's test, *p*<0.05). The heads of a newly moulted presoldier (left) and 4th instar larva (right) are shown. DDW, double-distilled water.

The external morphologies of the mandibles and heads of presoldiers were clearly different from those of the 4th instar larvae (electronic supplementary material, figure S5*a*,*b*). All DDW-injected No. 1 larvae differentiated into presoldiers by day 10.5±3.0 (mean ± s.d.) after appearance (electronic supplementary material, figure S5*c*; *n*=9). However, after the injection of DA receptor antagonist (*n*=20), 13 larvae differentiated into presoldiers (electronic supplementary material, figure S5*d*; day 10.8±2.4) and the remaining seven larvae moulted into 4th instars (electronic supplementary material, figure S5*e*; day 16.0±6.7). Note that dose-dependent effects of the antagonist were not observed (numbers of presoldiers and 4th instar larvae: 6 and 4 (100 pM), 7 and 3 (1 nM)) and, thus, the results were pooled. Presoldier differentiation rates of the antagonist-injected No. 1 larvae (65%) were significantly lower than those of the DDW-injected individuals (100%) (figure 2; Ryan's test, *p*<0.05).

#### 3.3.2 Behavioural observations

**Figure 3.**
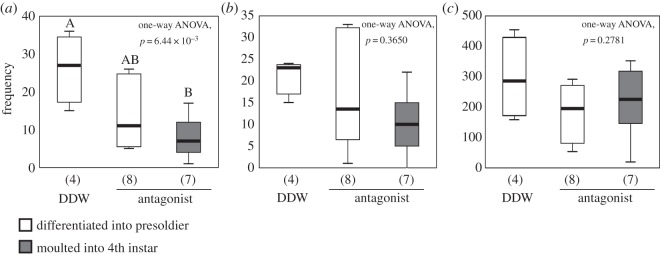
Behavioural observations in the presoldier- and 4th instar-destined larvae after treatments. The frequencies of proctodeal trophallaxis from reproductives (*a*), allogrooming toward reproductives (*b*) and locomotor activities (*c*) observed in the solution-injected No. 1 larvae. Numbers of colonies examined are shown in parentheses. The boxes and whiskers mean median, quartiles and range. The data are consistent with the use of parametric statistics by Levene's test (proctodeal trophallaxis: *p*=0.394; allogrooming: *p*=0.058; locomotor activities: *p*=0.675) prior to the use of the ANOVAs. Different letters above the columns indicate significant differences between categories (one-way ANOVA followed by Tukey's test, *p*<0.05). DDW, double-distilled water.

The frequencies of proctodeal trophallaxis from reproductives (both queen and king) to the 4th instar-destined larvae after injection of antagonist were significantly lower than those of the presoldier-destined larvae after injection of DDW (figure 3*a*; electronic supplementary material, movie S1; one-way ANOVA followed by Tukey's test, *p*<0.05). The same tendencies were observed when the frequencies of proctodeal trophallaxis from a queen or a king were counted separately (electronic supplementary material, figure S6; *p*<0.05). The frequencies of the allogrooming behaviour and locomotor activity observed in the No. 1 larvae were not significantly different among all cases (figure 3*b*,*c*; electronic supplementary material, movies S2 and S3; *p*=0.365 and 0.278).

## 4. Discussion

We focused on the ontogeny of the No. 1 larvae in incipient colonies of *Z. nevadensis* to examine the proximate mechanisms of presoldier differentiation. The results showed that the frequencies of proctodeal trophallactic behaviour from both reproductives to the No. 1 larvae were decreased by the injection of a DA receptor antagonist. Presoldier differentiation from the antagonist-treated No. 1 larvae was significantly suppressed, and normal larval moult (3rd instar to 4th instar) occurred alternately. This study showed the role of DA involved in caste polyphenism of social insects.

### 4.1 Different brain dopamine levels between No. 1 and No. 2 larvae

Brain DA levels of the No. 1 larvae at day 3 were significantly higher than those at day 0, but these tendencies were not observed in the No. 2 larvae. In animals, DA is mainly synthesized in nervous tissues and it binds to receptors expressed on various target cells [21]. In general, TH is known as the rate-limiting enzyme [38,39], and DDC is involved in the final steps of DA biosynthesis [40]. DA is well known to be involved in insect cuticular tanning, and biosynthetic genes are strongly expressed at epidermis [41,42]. In the No. 1 larvae, expression levels of these genes were high at day 0–1, but no significant differences were observed between days 2–3 and 4–5. On the other hand, although *TH* expression levels of the No. 2 larvae were high at day 0–1 and 2–3, *DDC* expression patterns of the No. 2 larvae were completely different from those of the No. 1 larvae. These differences may be related to the brain DA levels of the No. 1 and No. 2 larvae. The previous study showed that the proctodeal trophallactic behaviour of the No. 1 larvae was the most frequently observed at day 3 among 3rd instar stages examined [18]. However, those of the No. 2 larvae were consistently low for these periods. Moreover, allogrooming behaviours were more frequently observed in the No. 2 larvae compared with the No. 1 larvae during these periods. Therefore, there is a possibility that the different brain DA levels are related to behavioural differences observed in the No. 1 and No. 2 larvae. Soldiers possess a sclerotized head with pigmented cuticle, and specific cuticular formation occurs during soldier differentiation in *Reticulitermes speratus* [17] and *Hodotermopsis sjostedti* [43]. Consequently, the highly expressed genes in the No. 1 larvae (*TH*and *DDC*) could be also involved in soldier-specific cuticular formation in this species.

In invertebrates, DA is converted into NADA by NAT to inactivate DA in the CNS [20,44]. In the No. 1 larvae, brain NADA levels at day 3 were not significantly different from those at day 0, suggesting that inactivated DA levels in the brain were not increased during presoldier differentiation. This implication was consistent with the results of *NAT* expression patterns, which were essentially similar to those of *TH* and *DDC*. By contrast, in the No. 2 larvae, the brain NADA levels at day 3 were significantly higher than those at day 0, and constant *NAT* expression was observed for at least 5 days after appearance. In the Chinese tussah moth, *Antheraea pernyi*, *NAT* was involved in circadian rhythms via melatonin production and circadian gene expression [45]. Moreover, generally in insects, cuticular tanning is regulated by the expression of *NAT* as well as *TH* and *DDC* [46]. Further analyses should be performed to examine the possibility that high brain NADA levels or inactivation of DA are involved in specific behaviours (e.g. allogrooming) and cuticular formation observed in the No. 2 larvae.

### 4.2 Roles of dopamine during presoldier differentiation

Expression levels of *DopR1*–*3* genes in both No. 1 and No. 2 larvae were constant during the periods examined (electronic supplementary material, figure S4). These results indicated that receptor activity did not change substantially compared with DA synthesis gene expression levels (e.g. *TH* and *DDC* shown above) and brain DA levels. The drug used in this study (*cis*-(Z)-flupenthixol) particularly antagonized Dop1- and Dop2-like receptors, and repressed DA signalling in the brains of *A. mellifera* [47,48]. In the cockroach *P. americana*, an overdose of this drug acted weakly on the receptors of other amines, such as octopamine [49]. The concentrations applied in the present experiments (32.2 nl of 100 pM or 1 nM) were much lower than those used in other insects; 1 μl of 10 mM to *A. mellifera* abdomen [50] and 10 μl of 20 mM to *P. americana* haemolymph [22]. Because these previous studies showed no evidence of antagonism of the receptors of other amines, the present drug concentrations may have little or no effect on other receptors.

Among the three different behaviours examined, the frequency of the proctodeal trophallactic behaviour was only suppressed by treatment with the DA receptor antagonist, especially in the 4th instar larvae-destined individuals. These results supported the idea that repeated proctodeal trophallactic behaviour is important for presoldier differentiation, as proposed in a previous report [18]. The present results also suggested that these behaviours were strongly promoted by high brain DA levels and/or DA receptor signalling activity. There are some evidences that species-specific insect behaviours are observed depending on brain DA levels [22–26,51]. Because the action of the DA receptor antagonist gradually reduced with the time after the injection [48], repeated drug injection may have stronger effects on the reduction in the frequency of behaviours. Further analyses should be performed to confirm this suggestion. Moreover, artificial induction of DA or knockdown analyses of DA synthesis genes may also be effective methods to examine this issue.

DDW-treated No. 1 larvae always differentiated into presoldiers, but some drug-treated No. 1 larvae moulted into 4th instar larvae, suggesting that DA receptor signalling is required for presoldier differentiation in incipient colonies of this species. In *D. melanogaster*, DA may be a potent modulator to regulate JH titre by promoting the activity of the corpora allata [52]. JH–DA interactions were involved in locomotor activities and flight behaviours in male honeybees [30,50] and the carpenter bee *Xylocopa appendiculata* [53]. In *Z. nevadensis*, *JHAMT* and *CYP15A1* genes involved in the final steps of JH biosynthesis were highly expressed in the heads of No. 1 larvae at days 2–3 and 4–5 after their appearance, respectively [28]. On the other hand, the No. 1 larvae most frequently received proctodeal foods from reproductives at day 3 after their appearance, and the frequency of trophallaxis was gradually reduced afterwards [18]. Consequently, we hypothesize that DA signalling in the No. 1 larvae may affect the JH level through changes in behaviour and frequencies of trophallactic food intake and JH-dependent developmental formation. However, presoldier differentiation of the Formosan subterranean termite *Coptotermes formosanus* was not influenced by application of DA to workers, suggesting that feeding experiments using solvents of biogenic amines were limited to elucidate the functions of biogenic amines during presodier differentiation [54]. Future studies should be performed to elucidate the molecular relationships between DA and JH signalling during termite caste differentiation.

## 5. Conclusion

Proctodeal trophallactic behaviour is broadly observed among termite lineages. Various factors as social signals, such as nutrients and microbiota, are transferred from donors to recipients via trophallaxis [55]. Although the exact contents of trophallactic foods from reproductives are still unknown, nutrients, pheromone and/or JH itself may be transferred to the No. 1 larvae [18]. The present study showed that increased brain DA levels in the No. 1 larvae positively related to the repeated trophallaxis with reproductives and transmission of some social factors. This is the first study to highlight the role of DA during termite caste differentiation. DA role for caste differentiation should be investigated in other social insects with frequent trophallactic and allogrooming behaviour.

## Supplementary Material

Supplementary figures S1-S6

## Supplementary Material

Supplementary table 1.

## Data accessibility

Dryad (doi:10.5061/dryad.k9v42).
