# Supplementary figures and images for: Dopamine regulates termite soldier differentiation through trophallactic behaviours
Source: R Soc Open Sci. 2016 Feb 10;3(2):150574. doi: 10.1098/rsos.150574 (PMC4785978; doi:10.1098/rsos.150574)

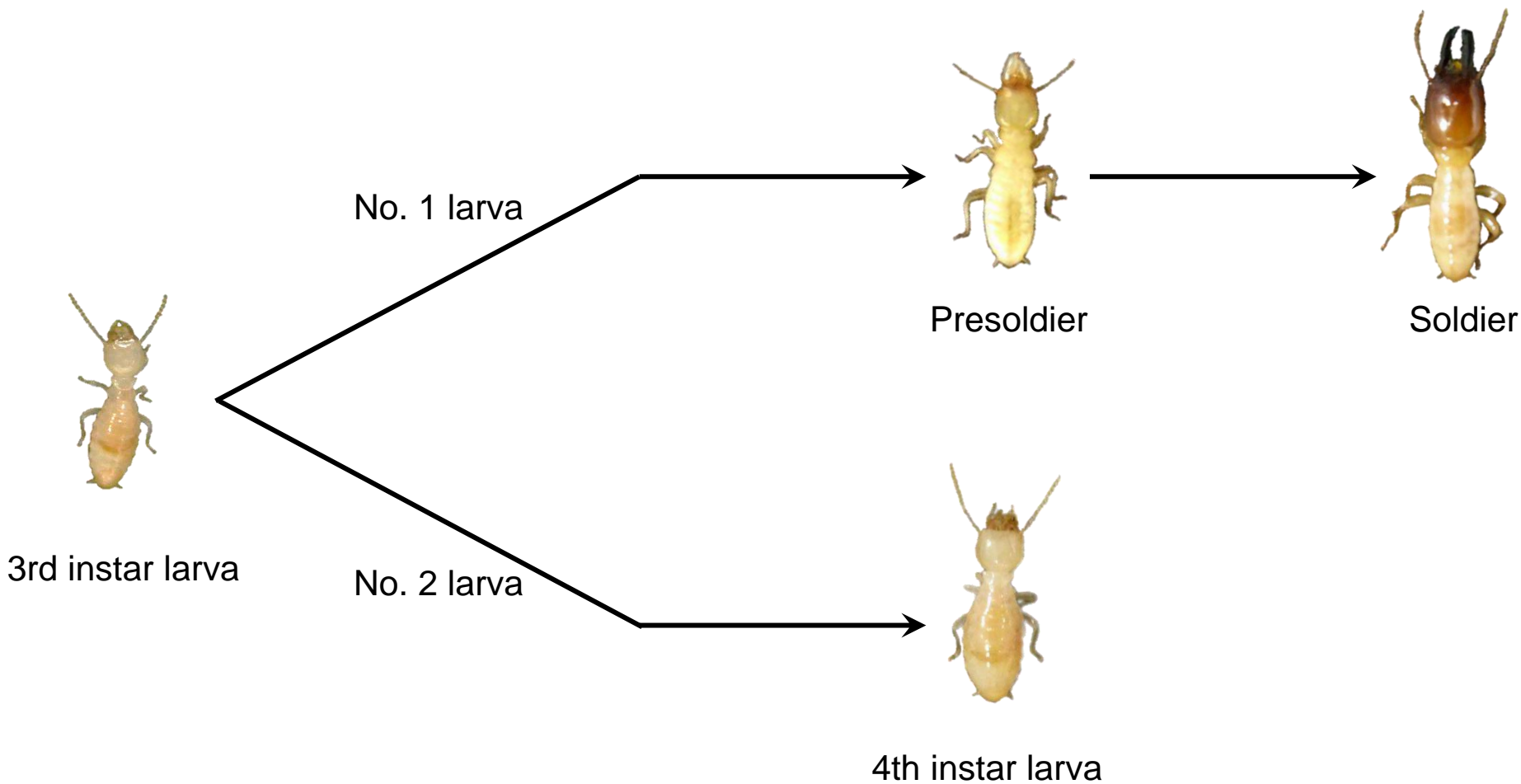

Figure S1

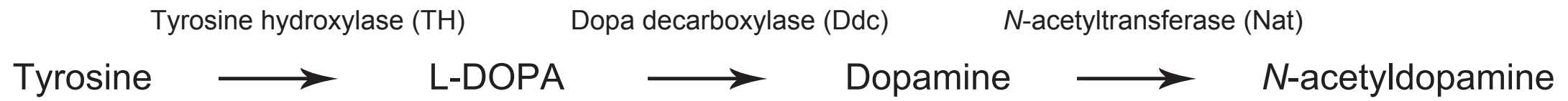

Figure S2

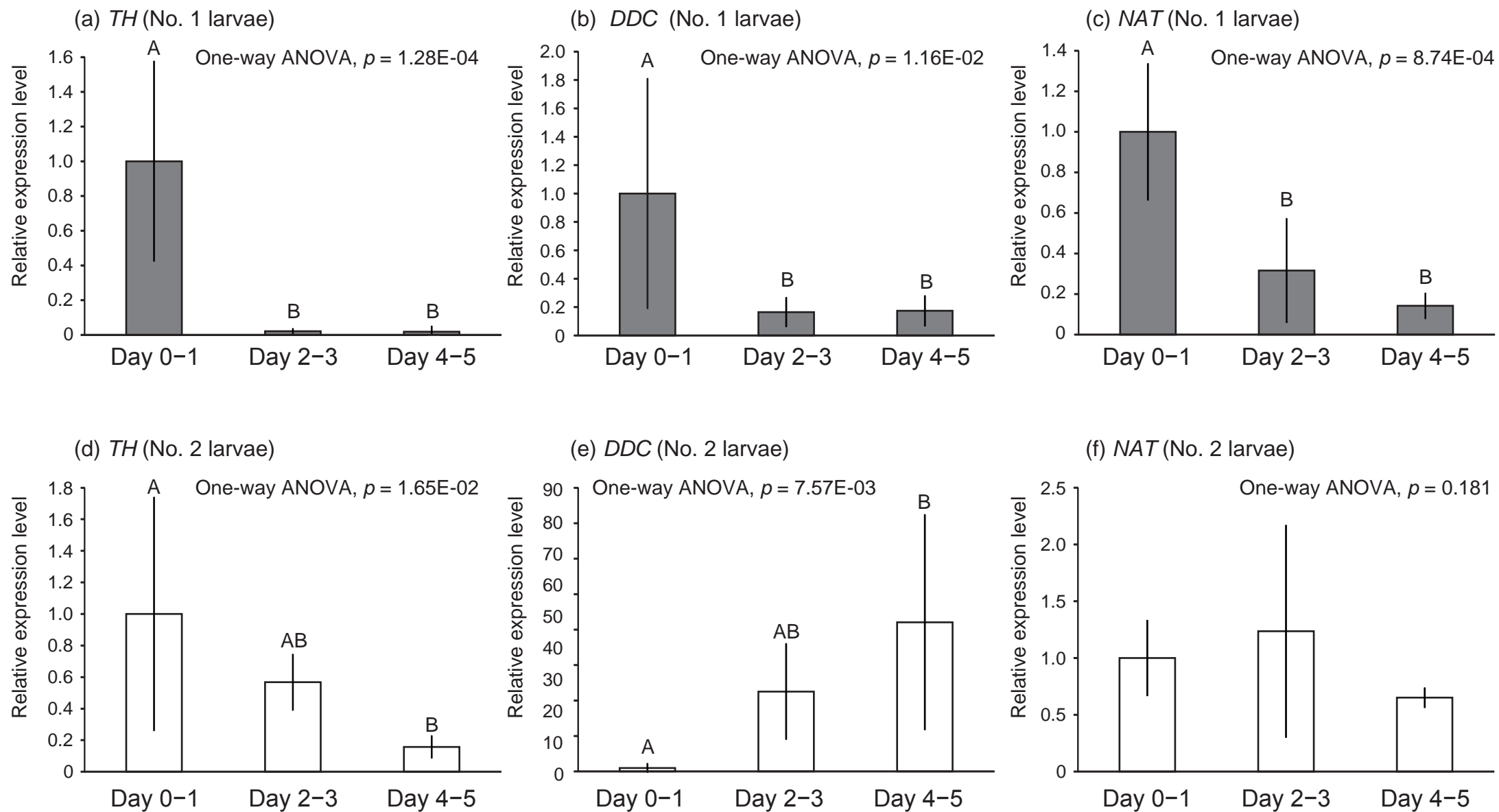

Figure S3

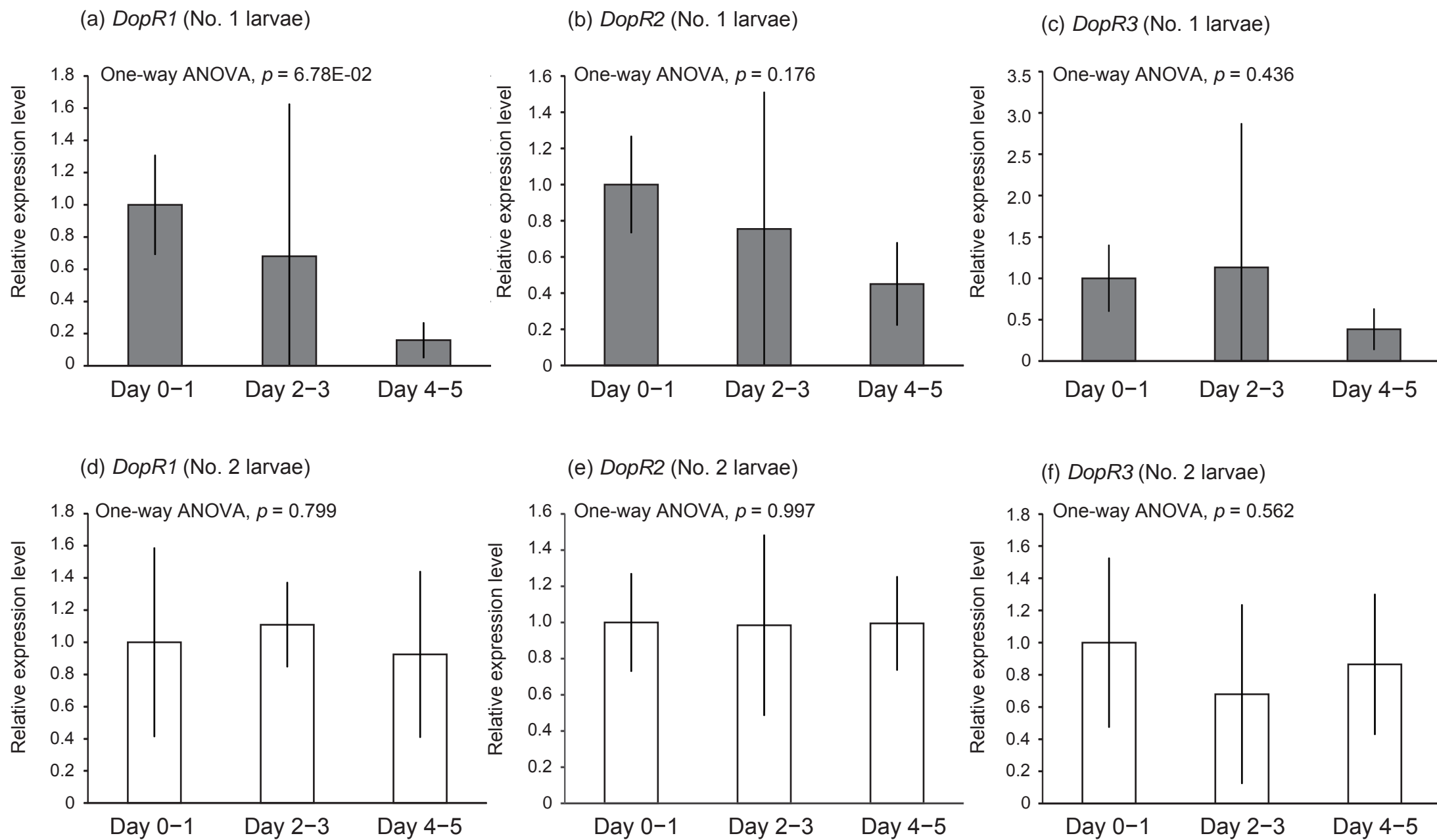

Figure S4

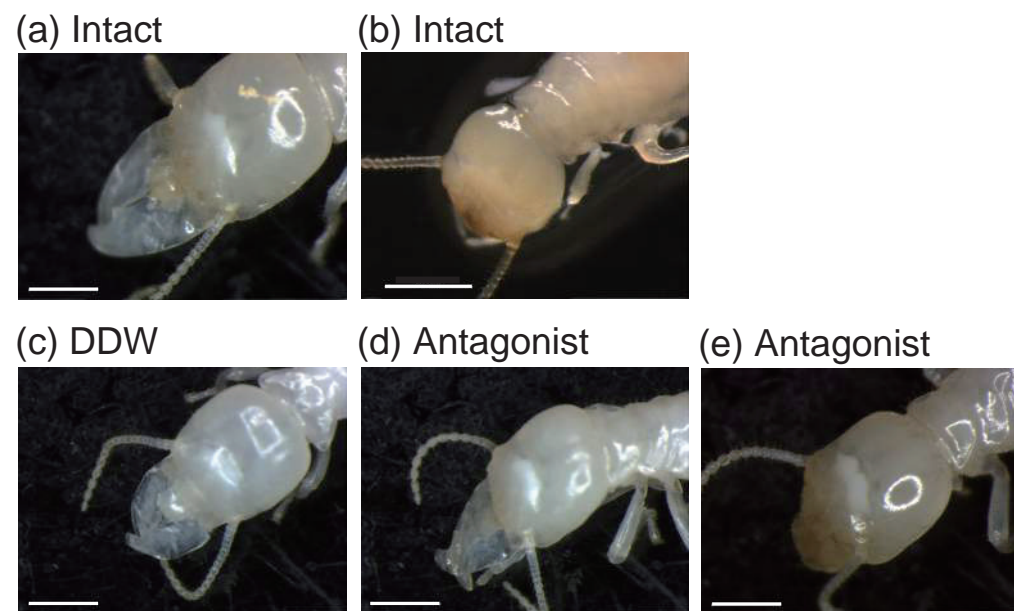

Figure S5

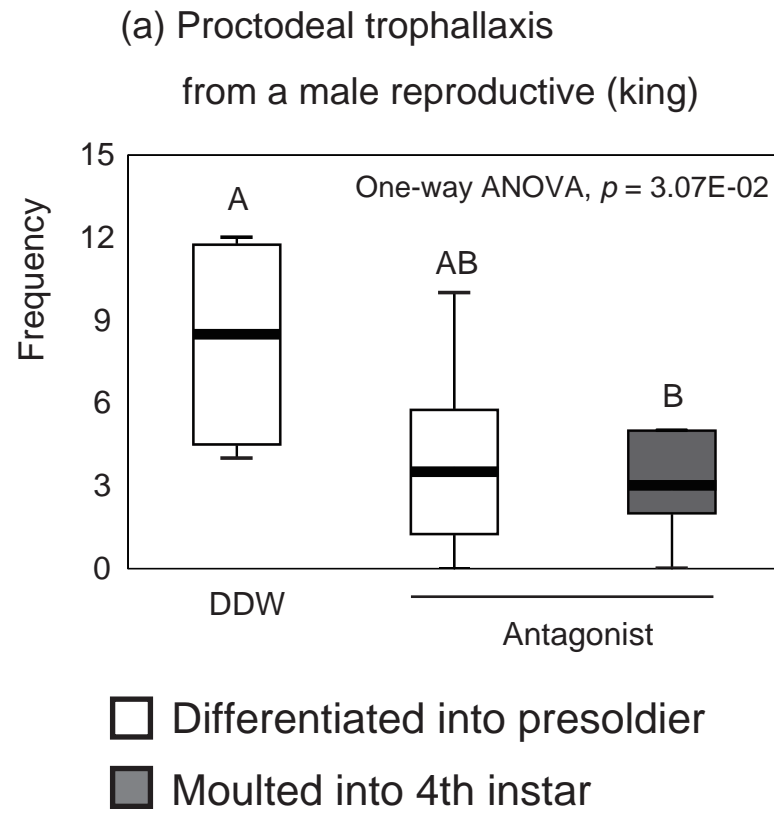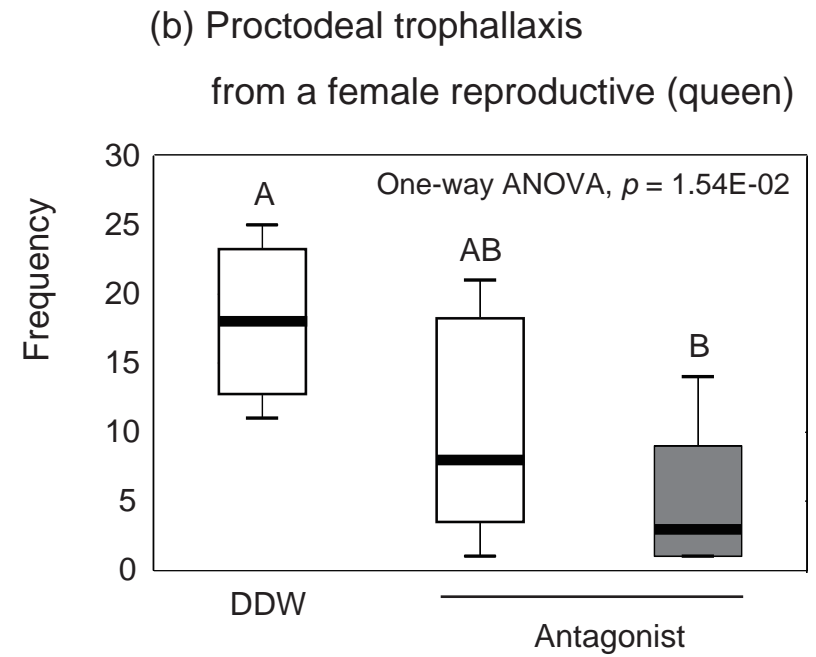

Figure S6

Supplement: Supplementary figures S1-S6 [file rsos150574supp1.pdf]
